# Epistatic Interaction between *BANK1* and *BLK* in Rheumatoid Arthritis: Results from a Large Trans-Ethnic Meta-Analysis

**DOI:** 10.1371/journal.pone.0061044

**Published:** 2013-04-30

**Authors:** Emmanuelle Génin, Baptiste Coustet, Yannick Allanore, Ikue Ito, Maria Teruel, Arnaud Constantin, Thierry Schaeverbeke, Adeline Ruyssen-Witrand, Shigeto Tohma, Alain Cantagrel, Olivier Vittecoq, Thomas Barnetche, Xavier Le Loët, Patrice Fardellone, Hiroshi Furukawa, Olivier Meyer, Benjamin Fernández-Gutiérrez, Alejandro Balsa, Miguel A. González-Gay, Gilles Chiocchia, Naoyuki Tsuchiya, Javier Martin, Philippe Dieudé

**Affiliations:** 1 Institut National de la Santé et de la Recherche Médicale UMR-S946, Univ Paris Diderot, Paris, France; 2 Rheumatology Department, Bichat Hospital, Assistance Publique Hôpitaux de Paris, Univ Paris Diderot, Paris, France; 3 Rheumatology Department A, Cochin Hospital, Assistance Publique Hôpitaux de Paris, Univ Paris Descartes, Paris, France; 4 Institut National de la Santé et de la Recherche Médicale UMRS-S1016, Univ Paris Descartes, Cochin Hospital, Paris, France; 5 Faculty of Medicine, University of Tsukuba, Tsukuba, Japan; 6 Instituto de Parasitologia y Biomedicina Lopez-Neyra, Consejo Superior de Investigaciones Científicas, Granada, Spain; 7 UMR 1027, Institut National de la Santé et de la Recherche Médicale, Toulouse III University and Rheumatology Department, Purpan Hospital, CHU Toulouse, Toulouse, France; 8 Rheumatology Department, Pellegrin Hospital, Bordeaux Selagen University, Bordeaux, France; 9 Clinical Research Center for Allergy and Rheumatology, Sagamihara Hospital, National Hospital Organization, Sagamihara, Kanagawa, Japan; 10 Rheumatology Department, CHU de Rouen-Hôpitaux de Rouen, Institut National de la Santé et de la Recherche Médicale U905, Institute for Research and Innovation in Biomedicine, Rouen University, Rouen, France; 11 Rheumatology Department, Amiens Teaching Hospital, University of Picardie - Jules Verne, Amiens, France; 12 Rheumatology Department, Hospital Clínico San Carlos, Madrid, Spain; 13 Hospital La Paz, Madrid, Spain; 14 Rheumatology Department, Hospital Marques de Valdecillas, Santander, Spain; 15 Institut National de la Santé et de la Recherche Médicale U699, Bichat Faculty of Medicine, Univ Paris Diderot, Paris, France; South Texas Veterans Health Care System and University Health Science Center San Antonio, United States of America

## Abstract

**Background:**

*BANK1* and *BLK* belong to the pleiotropic autoimmune genes; recently, epistasis between *BANK1* and *BLK* was detected in systemic lupus erythematosus. Although *BLK* has been reproducibly identified as a risk factor in rheumatoid arthritis (RA), reports are conflicting about the contribution of *BANK1* to RA susceptibility. To ascertain the real impact of *BANK1* on RA genetic susceptibility, we performed a large meta-analysis including our original data and tested for an epistatic interaction between *BANK1* and *BLK* in RA susceptibility.

**Patients and Methods:**

We investigated data for 1,915 RA patients and 1,915 ethnically matched healthy controls genotyped for *BANK1* rs10516487 and rs3733197 and *BLK* rs13277113. The association of each SNP and RA was tested by logistic regression. Multivariate analysis was then used with an interaction term to test for an epistatic interaction between the SNPs in the 2 genes.

**Results:**

None of the SNPs tested individually was significantly associated with RA in the genotyped samples. However, we detected an epistatic interaction between *BANK1* rs3733197 and *BLK* rs13277113 (*P_interaction_* = 0.037). In individuals carrying the *BLK* rs13277113 GG genotype, presence of the *BANK1* rs3733197 G allele increased the risk of RA (odds ratio 1.21 [95% confidence interval 1.04–1.41], *P* = 0.015. Combining our results with those of all other studies in a large trans-ethnic meta-analysis revealed an association of the *BANK1* rs3733197 G allele and RA (1.11 [1.02–1.21], *P* = 0.012).

**Conclusion:**

This study confirms *BANK1* as an RA susceptibility gene and for the first time provides evidence for epistasis between *BANK1* and *BLK* in RA. Our results illustrate the concept of pleiotropic epistatic interaction, suggesting that *BANK1* and *BLK* might play a role in RA pathogenesis.

## Introduction

Rheumatoid arthritis (RA) is a systemic, inflammatory, autoimmune disorder that affects up to 1% of the general adult population worldwide [1]. Nearly two-thirds of cases are seropositive for rheumatoid factor (RF) or anti-citrullinated protein autoantibodies (ACPA) [2]. RA is a complex disease implicating environmental, genetic and stochastic factors. However, the weight of genetics is underlined by family studies estimating that more than 50% of the variance in disease risk is explained by genetic factors [3]. To date, a genetic etiology for RA is unequivocal: recent genome-wide association studies have identified at least 31 validated susceptibility loci and implicated a broad array of biological pathways. These risk alleles explain about 16% of the disease variance, so additional susceptibility variants remain to be discovered [4].

Family-based epidemiology studies suggest a shared genetic basis among several autoimmune diseases leading to the paradigm of pleiotropy [5]. This genetic overlap is illustrated by the well-known association of certain human leukocyte antigen (*HLA*) loci and multiple human autoimmune diseases, as well as non-*HLA* risk loci in diverse pathways such as *PTPN22*, *STAT4*, *IRAK1* or *IRF5* (reviewed in [6]).

*BANK1*, encoding the B-cell scaffold protein with ankyrin repeats 1, is a Lyn tyrosine kinase substrate that promotes tyrosine phosphorylation of inositol 1,4,5-trisphosphate receptors [7]. *BANK1* has been convincingly identified as a risk factor for the autoimmune diseases systemic lupus erythematosus (SLE) and systemic sclerosis (SSc) [8]–[12]. However, in terms of the contribution of *BANK1* to RA susceptibility, case–control association studies of the European Caucasian population gave conflicting results [13]–[15]. The association signal for the human *BANK1* gene is located between exons 1 and 3, with 2 polymorphisms, rs17266594 and rs10516487, in very high linkage disequilibrium (LD) with each other [8], [14]. Recently, the rs17266594 SNP, located in the branch-point consensus sequence, was found to have a negligible effect on splicing or gene expression, whereas the non-synonymous SNP rs10516487 (R61H) showed a dual nature by first influencing mRNA splicing and then protein quantity and second producing a risk-variant-containing protein isoform with increased potential for multimerization [16]. A third non-synonymous SNP, rs3733197, (A383T) located in exon 7 and encoding the ankyrin repeat-like motif, was found strongly associated with both SLE and SSc [8]–[11].

*BLK* was also recently identified as a susceptibility gene for multiple autoimmune phenotypic traits, including SLE [9], [17], [18], SSc [19]–[21], Sjögren's syndrome [22] and RA [15], [23]–[25]. *BLK* is a Src tyrosine kinase specifically expressed in the B-cell lineage [26]. The pleiotropic genetic variant of *BLK*, rs13277113, is located in the promoter of *BLK*, and the risk genotypes may be associated with reduced *BLK* mRNA levels in SLE [17].

Thus, both *BANK1* and *BLK*, which encode for proteins involved in B-cell signaling, could be considered autoimmune pleiotropic genes. Of interest, a genetic and physical interaction of *BANK1* and *BLK* was recently detected in SLE [27]. We aimed to re-evaluate the role of *BANK1* in RA susceptibility and test whether *BANK1– BLK* epistatis could be involved in the RA genetic background, notably in the subset of patients positive for specific autoantibodies.

## Materials and Methods

### Patients and controls

A total of 1,454 RA patients (820 from France and 634 from Japan) and 1,542 ethnically matched healthy controls (1,220 from France and 322 from Japan) were genotyped for *BANK1* rs10516487 and rs3733197 and BLK rs13277113. These original data were combined with already published data for 461 cases and 373 controls from Spain genotyped for the same 3 SNPs [13], for a total of 1,915 RA cases and 1,915 ethnically matched healthy controls. All RA patients were tested for 1) anti-CCP2 antibodies detected by commercial ELISA (Immunoscan, Eurodiagnostica, Arnheim, The Netherlands), and 2) RF by laser nephelometry.

All subjects provided informed, written consent as approved by the institutional review boards for the recruiting sites of the affiliated institutions (CPP IDF8, Paris, France; ethics committee, University of Granada, Spain; and ethics committee, University of Tsukuba, Japan).

To ascertain the real impact of *BANK1* on RA susceptibility, we performed a meta-analysis including our original data and published data for 2,119 patients with RA and 2,163 controls from Spain, Sweden, Argentina, and Mexico [14]. For meta-analysis of *BANK1* rs10516487, we included 5,325 RA patients and 11,780 controls from the United Kingdom: 1) 3,468 RA cases and 8,847 controls described in [15] with genotype and clinical information kindly provided by Dr. Orozco and 2) 1,857 RA cases and 2,933 controls from the Wellcome Trust Case Control Consortium [28]. All patients fulfilled the American College of Rheumatology 1987 revised classification criteria for RA [29].

### Genotyping methods

Subjects were genotyped for *BANK1* rs10516487 and rs3733197 and *BLK* rs13277113 by use of a competitive allele-specific PCR system (Kaspar genotyping, Kbioscience, Hoddeston, UK) and TaqMan 5′ discrimination assay (Applied Biosystem, Foster City, SA).

### Statistical analysis

Statistical analyses involved use of R v2.13.1 (http://www.R-project.org, the R Foundation for Statistical Computing, Vienna, Austria). Tests of conformity to Hardy–Weinberg equilibrium involved a standard chi-square test (1 degree of freedom [df]) of differences between observed and expected genotype counts.

The association of SNPs and RA was tested by a logistic regression model assuming multiplicative effects (*i.e.*, with genotype AA as reference, the risk associated with the homozygous GG genotype was assumed to be the square of the risk associated with the AG genotype). To test for interaction between the alleles for the two SNPs, we used a multivariate logistic regression model, with the 2 main effects for each of the SNPs (assuming multiplicative effects of the alleles at each locus) and an interaction parameter. Analysis was performed for each sample separately, the 2 European Caucasian samples combined, and the whole sample (after adjusting for country of origin). Significance of the interaction parameter was tested by a 1-df likelihood-ratio test. The corresponding *P*-values for epistasis were termed *P_interaction_* and the odds ratio of the interaction parameter OR_interaction_. We performed a stratified analysis to estimate the ORs for *BANK1* rs10516487 and rs3733197 alleles assuming a multiplicative model for subsets of subjects carrying the AA, AG or GG genotypes of *BLK* rs13277113.

Another test of interaction involved the Model Based Multifactor Dimensionality Reduction (MB-MDR) method [30] with R package MBMDR-3.0.2 [31]. This method is a dimension reduction method for exploring gene–gene interactions that involves merging multilocus genotypes into a 1-D construct that reflects the risk and can be represented by 3 values: high, low and non-informative. The method consists of 3 steps. In the first step, an association test is performed for each multilocus genotype and, depending on the resulting p value, the mutilocus genotype is assigned to 1 of the 3 categories: “high” if the p value is ≤0.1 and the score is positive, “low” if the p value is ≤0.1 and the score is negative and “undetermined” if the p value is >0.1. In a second step, multilocus genotypes of the same risk category are merged and 2 new association tests are performed, one for the high-risk category and one for the low-risk category. To account for multiple testing, the significance of the maximum of these two Wald statistics is then assessed by permutations.

We performed a meta-analysis of the *BANK1* association with RA using the R package rmeta by pooling the results from the current study for *BANK1* and results from the literature by searching the Medline database using the following keywords: rheumatoid arthritis, gene, BANK1 [32]. The DerSimonian-Laird random effects model [33] was used for allele counts in the different samples. Heterogeneity between samples was tested by the Q test [34] and the I^2^ statistic [35]. This study complies with the PRISMA statement [36].

## Results

For both French and Japanese samples, the genotyping success rate was >95%, with no significant departure from Hardy–Weinberg equilibrium at p<0.05. In addition, control allelic frequencies for *BANK1* rs10516487 and rs3733197 and *BLK* rs13277113 were similar to those reported for the European Caucasian and Japanese populations in the HapMap project (http://hapmap.ncbi.nlm.nih.gov/).

We had available genotype data for the 3 markers for 1,901 RA patients (806 from France, 461 from Spain and 634 from Japan) and 1,898 ethnically matched controls. The characteristics of RA patients are in Table 1.

**Table 1:** Characteristics of rheumatoid arthritis cases investigated for a *BLK*–*BANK1* genetic interaction.

	France* (N = 806)	Spain (N = 461)	Japan* (N = 634)
**Sex ratio (M/F)**	0.31	0.34	0.24
**Age at disease onset**	43.07±14.79	48.17±16.15	48.47±13.69
**Disease duration**	11.28±7.92	15.45±6.71	15.11±10.62
**Rheumatoid factor** **	523 (70.1%)	347 (75.9%)	548 (86.6%)
**Anti-CCP antibodies** **	479 (64.7%)	300 (65.1%)	556 (90.7%)
**Erosive disease** **	445 (58.1%)	87 (78.4%)	358 (56.5%)

*Data are mean±SD or number (%) unless indicated.*

* *Genotype data for the 3 investigated single nucleotide polymorphisms were available for 1,440 of 1,454 RA cases from France and Japan.*

** *The percentage refers to percentage of positive patients among those with available data.*

**Figure 1:**
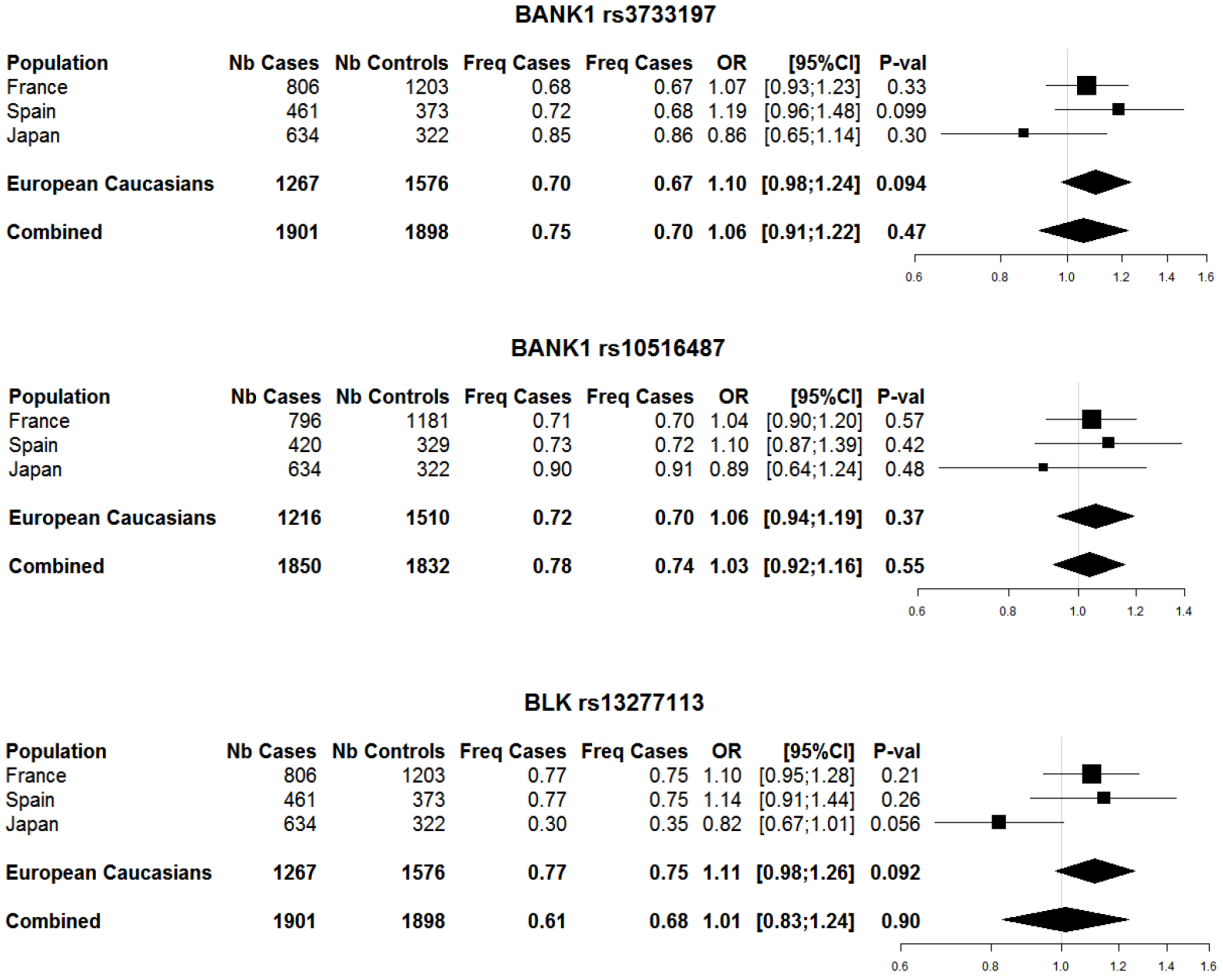
Results of single marker analysis in populations genotyped for both *BANK1* and *BLK*. Number of cases and controls, frequency of G allele, odds ratio (OR) and 95% confidence interval (95% CI) and *P*-value of the association under a multiplicative model for each population separately, for European Caucasians and for the combined dataset (France, Spain and Japan).

Analysis of single *BANK1* SNPs failed to detect an association with RA in each population investigated (*i.e.*, French, Spanish and Japanese) or the combined population (Figure 1). Similarly, we found no association of *BLK* rs13277113 and RA in all populations investigated (Figure 1).

**Figure 2:**
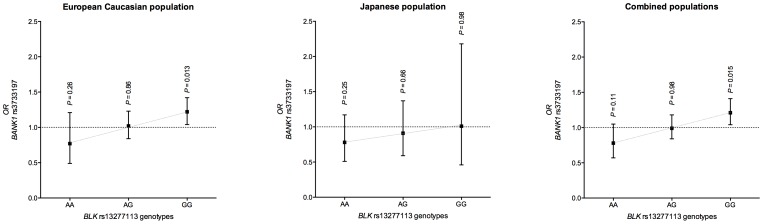
Epistasis between *BANK1* rs3733197 and *BLK* rs13277113. OR for trend for the G allele of *BANK1* rs3733197 and the three *BLK* rs13277113 genotypes by population (France, Spain and Japan).

We found an interaction between *BANK1* rs3733197 and *BLK* rs13277113 in the European Caucasian population (OR_interaction_ = 1.23 [95% confidence interval (95% CI) 1.02–1.48], *P_interaction_* = 0.032). The risk of RA was increased with the G risk allele of *BANK1* rs3733197 only for individuals carrying the GG homozygous genotype of *BLK* rs13277113 (OR_G/GG_ = 1.22 [1.04–1.42], *P* = 0.013). We found no effect of the rs3733197 G allele in subjects carrying the rs13277113 AG or AA genotypes (OR_G/AG_ = 1.02 [0.84–1.23], *P* = 0.86, and OR_G/AA_ = 0.77 [0.49–1.21], *P* = 0.26, respectively; Table 2, Figure 2). *BANK1* rs3733197 and *BLK* rs13277113 showed no epistasis in the Japanese population (OR_interaction_ = 1.15 [0.77–1.72], *P_interaction_* = 0.49). Meta-analysis of the combined sets (*i.e.*, French, Spanish and Japanese populations) confirmed the epistatic interaction between *BANK1* rs3733197 and *BLK* rs13277113 (OR_interaction_ = 1.17 [1.01–1.36], *P_interaction_* = 0.037), and results were homogeneous among the different populations (Q-test [2 df] = 0.85, *P* = 0.65, I^2^ = 0%). Concordant with results observed in the European Caucasian population, for the combined set, the association of the G risk allele of *BANK1* rs3733197 and RA was restricted to subjects carrying the *BLK* rs13277113 GG genotype (OR_G|GG(meta)_ = 1.21 [1.04–1.41], *P* = 0.015; Table 2, Figure 2).

**Table 2:** Epistasis between *BANK1* rs3733197 and *BLK* rs13277113 in rheumatoid arthritis: association of BANK rs3733197 G allele and BLK rs13277113 genotypes.

Sample	N cases	N controls	Freq Cases	Freq Controls	OR [95%CI]	*P-value*
***BLK*** ** rs13277113 GG genotype**						
France	479	674	0.697	0.667	1.16 [0.96–1.39]	*0.110*
Spain	275	213	0.722	0.662	**1.37 [1.03–1.84]**	***0.033***
European Caucasian	754	887	0.706	0.666	**1.22 [1.04–1.42]**	***0.013***
Japan	59	41	0.830	0.829	1.01 [0.46–2.18]	*0.981*
Combined*	813	928	0.715	0.673	**1.21 [1.04–1.41]**	***0.015***
***BLK*** ** rs13277113 AG genotype**						
France	280	458	0.659	0.666	0.97 [0.78–1.21]	*0.784*
Spain	162	132	0.738	0.708	1.15 [0.81–1.64]	*0.439*
European Caucasian	442	590	0.588	0.675	1.02 [0.84–1.23]	*0.861*
Japan	268	142	0.862	0.873	0.91 [0.59–1.37]	*0.663*
Combined*	710	732	0.753	0.714	0.99 [0.84–1.18]	*0.981*
***BLK*** ** rs13277113 AA genotype**						
France	47	71	0.649	0.669	0.91 [0.53–1.59]	*0.752*
Spain	24	28	0.604	0.750	0.52 [0.22–1.18]	*0.129*
European Caucasian	71	99	0.634	0.692	0.77 [0.49–1.21]	*0.260*
Japan	307	139	0.837	0.867	0.78 [0.51–1.17]	*0.248*
Combined*	378	238	0.799	0.794	0.78 [0.57–1.05]	*0.107*

Freq: frequency of the *BANK1* rs3733197 G risk allele, OR [95%CI]: odds ratio, 95% confidence interval.

* France, Spain and Japan combined.

The MB-MDR test for *BANK1* rs3733197 and *BLK* rs13277113 also provided significant results for the European samples (France and Spain combined and adjusted on country of origin) leading to a Wald statistics maximum (Wmax) of 9.44 for the high-risk GG-GG genotype and a *P*-value of 0.036 (assessed with 500 permutations) with 95% CI 0.019–0.052. No findings were significant for the Japanese sample, and the GG-GG genotype combination was not classified as high risk (Table 3).

**Table 3:** Distribution of BLK-rs13277113 and BANK1-rs3733197 multilocus genotypes in cases and control populations from Spain, France and Japan.

Country	*BLK* rs13277113	*BANK1* rs3733197	Cases	Controls	Beta	P-value	Risk category
Spain	AA	AA	3	3	−0.21	0.79	0
	AG	AA	12	13	−0.30	0.46	0
	GG	AA	14	20	−0.59	0.09	L
	AA	AG	13	8	0.28	0.54	0
	AG	AG	61	51	−0.04	0.85	0
	GG	AG	125	104	−0.04	0.81	0
	AA	GG	8	17	−0.99	0.02	L
	AG	GG	89	68	0.07	0.69	0
	GG	GG	136	89	0.28	0.07	H
France	AA	AA	8	6	0.69	0.20	0
	AG	AA	37	50	0.10	0.64	0
	GG	AA	43	63	0.02	0.92	0
	AA	AG	17	35	−0.33	0.27	0
	AG	AG	117	206	−0.19	0.12	0
	GG	AG	204	323	−0.07	0.44	0
	AA	GG	22	30	0.09	0.74	0
	AG	GG	126	202	−0.08	0.49	0
	GG	GG	232	288	0.25	0.01	H
Japan	AA	AA	5	4	−0.45	0.49	0
	AG	AA	10	1	1.64	0.12	0
	GG	AA	0	2	−2.29	0.23	0
	AA	AG	90	29	0.51	0.02	H
	AG	AG	54	34	−0.24	0.30	0
	GG	AG	20	10	0.02	0.97	0
	AA	GG	212	106	0.02	0.87	0
	AG	GG	204	107	−0.05	0.74	0
	GG	GG	39	29	−0.41	0.11	0

Number of cases and controls, beta coefficient for logistic regression, corresponding p-value and risk category (“H” for high risk, “L” for low risk and “O” for unclassified) from Model Based Multifactor Dimensionality Reduction analysis for each multilocus genotype.

We found no significant interaction of *BANK1* rs10516487 and *BLK* rs13277113, whatever the population investigated (data not shown).

**Figure 3:**
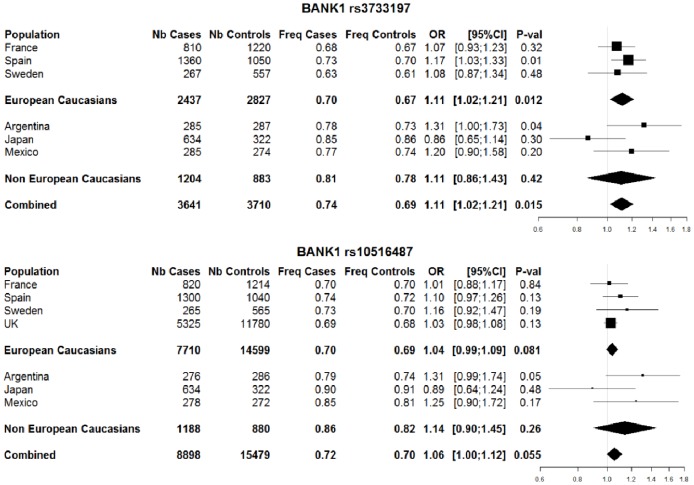
Meta-analysis of association of *BANK1* alleles and rheumatoid arthritis. Number of cases and controls; frequency of the G allele in cases and controls; and OR, 95% CI and *P*-value for the association for each population separately and for European Caucasians and non-European Caucasians and the combined dataset. All data were obtained from the literature, except for the France and Japan data, for which new genotyping was performed for this study.

The systematic review retrieved 2 studies [14], [15] (Figure S1). In combining our results with those of all association studies of the two *BANK1* SNPs and RA in a large trans-ethnic meta-analysis, we found an association of the functional *BANK1* rs3733197 polymorphism and RA, with the G allele conferring increased risk (OR = 1.11, [1.02–1.21], *P* = 0.012) (Figure 3). Similar ORs, although not significant, were obtained for non-European Caucasian populations (OR = 1.11 [0.86–1.43]; *P* = 0.42), probably because of smaller sample sizes than for the European Caucasian populations. The power (at p<0.05) to detect the effect of the G risk allele of BANK1 rs3733197 in the non-European populations was only 21.8% as compared with 60% for the European Caucasian populations. Effects were homogenous between all samples (Q-test = 5.97, *P* = 0.314; I^2^ = 16.3% [0%–78.8%]). Case–case analyses failed to detect any association with specific autoantibody status (ACPA and RF) (data not shown). Meta-analysis of the *BANK1* rs10516487 variant (R61H), which is functional [15], remained inconclusive, with an estimated OR of 1.06 ([1.00–1.12], P = 0.055; I^2^ = 13% [0%–74.6%]) for the association of the G allele and RA risk. The results were similar when restricting the analysis to European Caucasian populations or non-European Caucasian populations.

## Discussion

This study provides the first evidence of a contribution of the *BANK1* gene to RA susceptibility. It suggests that *BANK1* does not play a role in RA individually but only when interacting with *BLK*. In addition, this is the first report providing some evidence of epistasis between two RA susceptibility genes.

*BLK* has been convincingly established as an RA susceptibility gene, but the role of *BANK1* was uncertain: results of a previous meta-analysis were inconclusive and prompted us to investigate ethnic groups not investigated in the first meta-analysis [13]. Although we failed to detect any significant individual association for the two *BANK1* SNPs we investigated, the large trans-ethnic meta-analysis provided evidence for an association of *BANK1* rs3733197 and RA (OR = 1.11, [1.02–1.21], *P* = 0.015) (Figure 3).

The clustering of multiple autoimmune disorders in families and evidence for autoimmune pleiotropic loci are well known. With evidence for an association of *BANK1* and RA, both *BANK1* and *BLK* should be considered pleiotropic genes involved in the genetic background of distinct autoimmune phenotypes including SLE, SSc, Sjögren's syndrome and now RA. Interestingly, a genetic interaction between *BANK1* and *BLK* was recently identified in SLE [27]. Although the *BANK1–BLK* epistasis previously described in SLE involves different variants than those we investigated, our results strengthen the concept of pleiotropic genes and, even more importantly, pleiotropic genetic interactions pointing to a B-cell specific pathway that could be relevant in both SLE and RA pathogenesis.

*BLK* encodes a tyrosine kinase involved in the regulation of B-cell activation, and *BANK1* encodes for a B-cell adaptor protein [7], [37]. B lymphocytes play several critical roles in the pathogenesis of RA. They are the source of RF and ACPA and are involved in antigen presentation to T cells and cytokine production. The success of B-cell depletion therapy in RA illustrates the importance of this specific lymphocyte compartment [38]. Despite lack of functional or mechanistic data, an important limitation of this study, our results revealed an epistasis between a functional *BLK* variant and a *BANK1* non-synonymous variant (A383T) in exon 7, which encodes the ankyrin repeat-like motif [8], [17]. Consequently, both *BANK*1 and *BLK* disease-associated variants could contribute to sustained B-cell receptor signaling and B-cell hyperactivity. Further genetic association studies, involving large samples, are required to replicate our findings and better specify the putative role of *BANK1–BLK* epistasis in modulating the autoantibody status of RA, although we did not detect an association related to a particular ACPA status.

Of interest, Castillejo-López *et al.* recently demonstrated that B-cell receptor stimulation enhanced BANK1 and BLK binding [27], so genetics may guide the identification of true functional biological pathways in complex diseases [39]. Saijo *et al.* showed that mice triple deficient in the Src-family protein tyrosine kinases BLK and LYN but not single-deficient or SYK-deficient mice showed impaired NF-kappaB induction and B-cell development [40]. BANK1 contains several predicted motifs for binding the SH2 and SH3 domains of Src kinases. Therefore, *BANK1–BLK* epistasis may have specific functional consequences for B cells, which might be involved in RA pathogenesis.

In our samples of about 2,000 cases and 2,000 controls, we found no association of *BANK1* or *BLK* and RA but rather a significant genetic interaction. Taking into account the growing evidence that epistasis contributes to risk for complex diseases [41]–[43], our findings illustrate that accounting for gene–gene interactions might be necessary to identify genetic effects that could otherwise be missed. Indeed, the univariate approach considering only a single marker at a time, commonly used in genome-wide association studies, could overlook the complex interactions that often occur in biological systems [44].

Unexpectedly, the *BANK1–BLK* epistasis we identified involves the *BLK* rs13277113 GG genotype found protective in SLE. Such an inverse association could be surprising, but there are several examples of variants having opposite effects in distinct auto-immune diseases [45]. Another striking feature of the data is the huge difference in *BLK* rs13277113 minor allele frequency between the European samples and the Japanese sample. The G allele is indeed the major allele in Europe and the minor allele in Japan. Because of this difference, which could be due to differential selection between populations of different ancestry, use of the Japanese population as a replication sample to assess the *BANK1–BLK* epistasis detected in the European samples is difficult. Indeed, in terms of the multilocus genotype distribution in the Japanese sample (Table 3), the frequency of the GG-GG genotype combination was similar in cases and controls. However, the OR for the G allele of *BANK1* rs3733197 was increased for the GG genotype as compared with the AG and AA genotypes of *BLK* rs13277113. Consequently, independent replication is mandatory before definitely establishing the contribution of *BANK1–BLK* epistasis to RA susceptibility. Detecting and replicating true epistasis has been difficult and represents a challenge in deciphering the genetic architecture of complex diseases [41], [46].

In summary, *BANK1* contributes to the RA genetic susceptibility background, but the association may depend on the *BLK* rs13277113 genotype, in that our results suggest an epistasis between *BANK1* and *BLK*. This epistasis could explain the discrepancies in the various reported association studies and suggest that *BANK1* rs3733197 status should be re-analyzed for *BLK* rs13277113 status. Our findings illustrate the concept of pleiotropic epistatic interaction and suggest that BANK1 and BLK might play a role in RA pathogenesis.

## Supporting Information

Figure S1 **Prisma Flow diagram summarizing how the meta-analysis was conducted.**
(DOCX)
