# Supplementary material for: Epistatic Interaction between BANK1 and BLK in Rheumatoid Arthritis: Results from a Large Trans-Ethnic Meta-Analysis
Source: PLoS One. 2013 Apr 30;8(4):e61044. doi: 10.1371/journal.pone.0061044 (PMC3639995; doi:10.1371/journal.pone.0061044)
Supplement: Figure S1 — Prisma Flow diagram summarizing how the meta-analysis was conducted. (DOCX) [file pone.0061044.s001.docx]

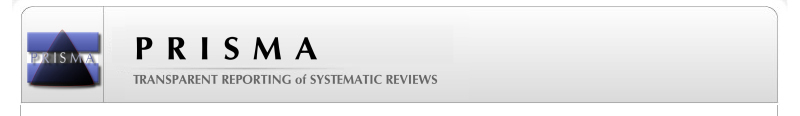
**PRISMA 2009 Flow Diagram**

Studies included in quantitative synthesis (meta-analysis)
(n = 2 )

Studies included in qualitative synthesis
(n = 2 )

Full-text articles excluded, with reasons
(n = 0 )

Full-text articles assessed for eligibility
(n = 2 )

Records excluded
(n = 4)

Records screened
(n = 6 )

Records after duplicates removed
(n = 6 )

Additional records identified through other sources
(n = 1 )

## Identification

## Eligibility

## Included

## Screening

Records identified through database searching
(n = 5 )
